# Contrast-Enhanced Magnetic Resonance Cholangiography: Practical Tips and Clinical Indications for Biliary Disease Management

**DOI:** 10.1155/2017/2403012

**Published:** 2017-02-28

**Authors:** Stefano Palmucci, Federica Roccasalva, Marina Piccoli, Giovanni Fuccio Sanzà, Pietro Valerio Foti, Alfonso Ragozzino, Pietro Milone, Giovanni Carlo Ettorre

**Affiliations:** ^1^Department of Medical and Surgical Sciences and Advanced Technologies-Radiodiagnostic and Radiotherapy Unit, University Hospital “Policlinico-Vittorio Emanuele”, 95123 Catania, Italy; ^2^UOC Diagnostica per Immagini PO “Santa Maria delle Grazie”, ASL NA2 Nord, Pozzuoli, Napoli, Italy; ^3^Diagnostica per Immagini S.R.L., Viale XX Settembre 53, 95129 Catania, Italy

## Abstract

Since its introduction, MRCP has been improved over the years due to the introduction of several technical advances and innovations. It consists of a noninvasive method for biliary tree representation, based on heavily T2-weighted images. Conventionally, its protocol includes two-dimensional single-shot fast spin-echo images, acquired with thin sections or with multiple thick slabs. In recent years, three-dimensional T2-weighted fast-recovery fast spin-echo images have been added to the conventional protocol, increasing the possibility of biliary anatomy demonstration and leading to a significant benefit over conventional 2D imaging. A significant innovation has been reached with the introduction of hepatobiliary contrasts, represented by gadoxetic acid and gadobenate dimeglumine: they are excreted into the bile canaliculi, allowing the opacification of the biliary tree. Recently, 3D interpolated T1-weighted spoiled gradient echo images have been proposed for the evaluation of the biliary tree, obtaining images after hepatobiliary contrast agent administration. Thus, the acquisition of these excretory phases improves the diagnostic capability of conventional MRCP—based on T2 acquisitions. In this paper, technical features of contrast-enhanced magnetic resonance cholangiography are briefly discussed; main diagnostic tips of hepatobiliary phase are showed, emphasizing the benefit of enhanced cholangiography in comparison with conventional MRCP.

## 1. Introduction

Magnetic Resonance Cholangiopancreatography (MRCP) provides high diagnostic accuracy in the assessment of biliary disease. It consists of a noninvasive method for biliary tree representation—based on heavily T2-weighted images in which stationary and slow-moving fluids in the bile ducts appear hyperintense in contrast to the hypointensity of the surrounding tissue [1–8]. Since its introduction, in 1986, several studies have been published describing technical advances and innovations acquired over the years [3, 6, 9–11].

Conventional MRCP includes two-dimensional (2D) sequences acquired with a radial thick-slab imaging or with coronal thin acquisitions oriented toward the hepatic hilum. In recent years, three-dimensional (3D) sequences have been added to the conventional protocol, increasing the possibility of biliary anatomy demonstration and leading to a significant benefit over conventional 2D imaging [7, 12].

Thanks to 2D and/or 3D sequences, conventional MRCP plays an important role in the diagnosis and characterization of several diseases. In the assessment of biliary lithiasis, it offers high diagnostic capability in comparison with other methods [13]. MRCP shows diagnostic accuracy comparable to that of ERCP in the evaluation of benign and malignant stenosis of the extrahepatic biliary tree [14]; in addition, it demonstrates high diagnostic capability in locating hepatolithiasis and detecting accompanying biliary strictures [15], in the identification of clinical alterations after cholecystectomy [16], in the assessment of normal or pathological changes after biliary surgery (hepatic resection, liver transplantation) [17, 18]. In all these mentioned clinical entities, MRCP—based on heavily T2-weighted sequences—provides a morphologic assessment.

Gadolinium liver-specific contrasts are cleared by glomerular excretion and biliary excretion; using gadoxetic acid, the percentage of elimination is about 50% for each route [19–21], whereas gadobenate dimeglumine is mainly excreted by the kidneys (up to 95%–97%). Several studies have been published since the early 1990s to better investigate the potential role of gadoxetic acid as a biliary contrast medium in MRI [22–24]. The biliary tree demonstration—obtained in the excretory phase using T1-weighted gradient echo images—offers the possibility of a new “functional phase” and/or “excretory phase” in a MRCP protocol.

Thus, the role of hepatobiliary contrast-enhanced Magnetic Resonance Cholangiography (MRC) is discussed in this article, exploring technical adjustments of imaging protocol; an overview of the most important clinical applications and tips is briefly reported, in order to help radiologists and nonradiologists in the management of biliary diseases.

## 2. Contrast-Enhanced Magnetic Resonance Cholangiography: Technical Features

3D interpolated T1-weighted spoiled gradient echo images have been proposed for the evaluation of the biliary tree after hepatobiliary contrast agent administration [25, 26]. These sequences are generally acquired in axial and/or coronal planes, using different techniques that depend on the scanner type.

**Figure 1:**
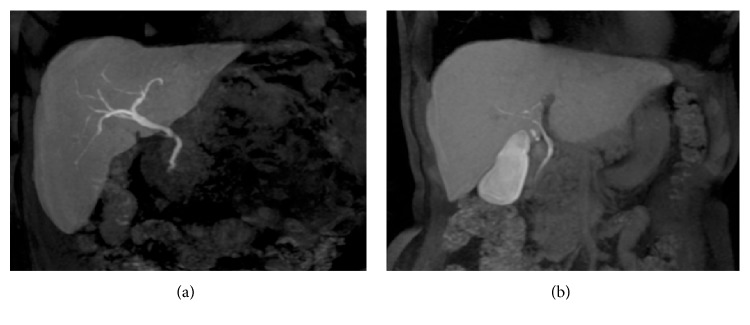
Gadolinium-enhanced MRC. MIP images of 3D T1-weighted spoiled gradient echo. (a) shows opacification of biliary tract after Gd-EOB-DTPA administration; hepatobiliary phase could be obtained 20 minutes after intravenous contrast. In a different patient, (b) demonstrates excretory phase obtained after gadobenate dimeglumine intravenous administration. In this case, the elimination of contrast media is provided by the kidney (95–97%) and the biliary system (2–5%): a satisfactory opacification of the biliary tree—in patients with preserved biliary function—is observed at least 60 minutes after contrast administration.

Biliary opacification (Figure 1) could be obtained using both hepatobiliary contrasts: gadobenate dimeglumine [27] or gadoxetic acid [12, 21].

Gadobenate dimeglumine has both an extracellular phase and a hepatobiliary phase; it has high relaxivity, with a weak transient albumin binding [28, 29]; only a small percentage is excreted into the bile (3–5%). The dose for its administration is 0.1 mmol/kg.

Gadoxetic acid has different pharmacokinetic properties: it is generally excreted in a percentage of 50% and 50%, respectively, by the liver and by the kidneys; in the liver, mechanisms of excretion involve multidrug resistance-associated proteins (MRPs) [30]. When hepatobiliary function is preserved, maximum enhancement in the hepatic parenchyma is reached 20 minutes after its administration, whereas gadobenate dimeglumine needs at least 60 minutes.

Thus, the choice of contrast agent influences the time for the acquisition of “excretory phase.”

**Figure 2:**
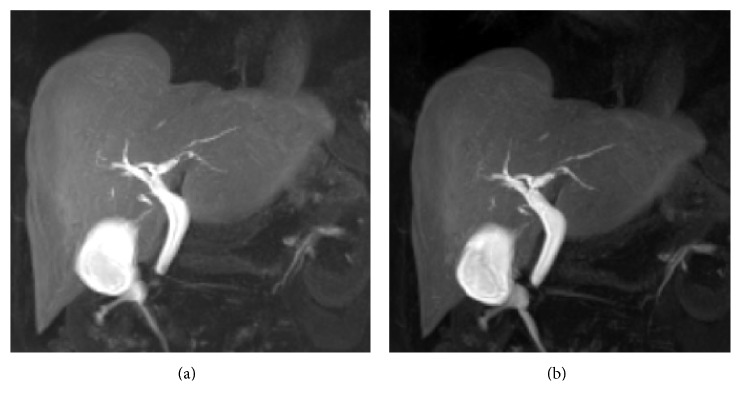
Influence of flip angles. 3D T1-weighted spoiled gradient echo sequences, with different flip angles (10° in (a), 30° in (b)). Delineation of the biliary ducts is better increasing the flip angle, depicted in (b) as well. The increase of flip angle improves the SNR and the CNR; a flip angle >20° is generally recommended.

Increased flip angle is recommended in the evaluation of the biliary tree using liver-specific gadolinium-enhanced MRC. Indeed, this flip angle variation influences the quality of hepatobiliary acquisitions (Figure 2); signal intensities derived from hepatic parenchyma, bile duct, and muscles have been measured on hepatobiliary phases acquired 20 minutes after gadoxetic acid administration, with variable flip angles [26]. Maximum intensities for biliary structures were registered with a FA of 35–40°, whereas the highest liver signal intensity was achieved with a FA of 25–30° [25].

In a recent paper by Stelter et al. [31], late hepatobiliary phases acquired with different flip angles—ranging from 10° to 35°—have been compared for the evaluation of the biliary tree. Delineation of biliary branches was better assessed using increased flip angle (35°) in gadolinium-enhanced MRC sequences. However, no statistical difference was reported for the mentioned kinds of acquisitions [31].

In another study, Stelter et al. [32] compared 3D TSE T2-weighted sequences and late T1-weighted acquisitions obtained after gadoxetic acid administrations [32]; T1 images were acquired with a flip angle of 35°. Late phase using T1-weighted acquisitions with a flip angle of 35° showed a good delineation of the entire biliary tree, whereas 3D TSE sequences reported lower scores. Depiction of intra-hepatic biliary ducts were better assessed using late enhanced phases; namely, in the evaluation of biliary variants, a certain degree of discordance was found (12.5%) and late MRC with gadoxetic acid showed higher confidence level than 3D TSE T2 acquisitions [32].

The importance of flip angle variation has been reported also in a paper by Frydrychowicz et al. [33], which analyzed a group of 10 healthy volunteers. Using gadoxetic acid, “optimal FA” of hepatobiliary phases were 25–30° and 45° for relative contrast liver versus muscle and relative contrast liver versus biliary structures, respectively; with gadobenate dimeglumine, “optimal FA” were 25–30° and 20° for relative contrast liver versus muscle and relative contrast liver versus biliary structures, respectively [33].

## 3. Contrast-Enhanced Magnetic Resonance Cholangiography: Clinical Indications

### 3.1. Anatomical Variations of the Biliary Tree

Anatomical variations of the biliary tree are common (about 30% of patients) [25], and knowledge regarding them is very important to avoid surgical complications [34, 35]. Conventional MRCP—based on 2D thick-slab acquisition—could be limited in the anatomical demonstration of variants: radiologists only suppose their insertions on thick-slab cholangiographic sequences. 3D T2-weighted biliary imaging improves visualization, thanks to the possibility of MPR, leading to an easier interpretation of the anatomy.

The biliary tree can be well depicted in the hepatobiliary phase MRI after administration of hepatobiliary contrast. Fat-suppressed 3D T1-weighted axial images are acquired with thin thickness, allowing MIP reformations [34].

**Figure 3:**
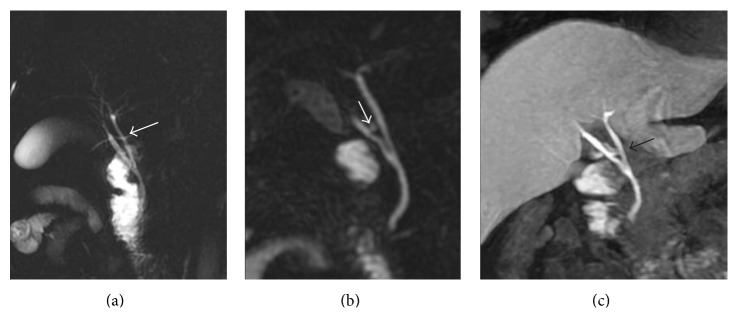
Anatomical variations of the biliary tree. 2D thick-slab cholangiography and 3D MIP FRFSE ((a) and (b), resp.), show a caudal confluence between right and left biliary ducts; in addition, an aberrant right duct is suspected on (a) and (b) (white arrow). Gadoxetic acid-enhanced MRC clearly demonstrates the right aberrant duct and the cystic duct (black arrow), with a separate insertion along left biliary duct.

**Figure 4:**
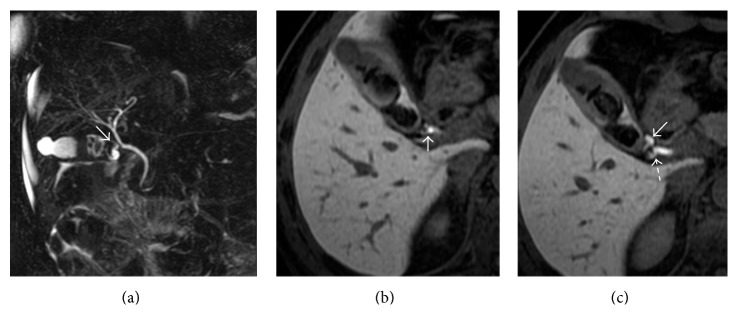
Anatomical variations of the biliary tree. MIP image of 3D FRFSE cholangiography shows multiple stones in the gallbladder (a). Axial 3D fast spoiled gradient echo images ((b) and (c)), obtained in an excretory phase, show right aberrant duct (white arrow in (b) and (c)) and the insertion of cystic duct (white dashed arrow).

Gd-EOB-DTPA-enhanced hepatobiliary phase MRI is a useful tool to evaluate biliary tree variations—also providing additional information as regards biliary flow, as referred by Hyodo et al. [34] (Figures 3 and 4).

Gd-EOB-DTPA-enhanced MR imaging is characterized by a higher SNR in the bile duct than conventional T2-weighted MRCP, with a better visualization of bile duct anatomy, especially in the intrahepatic bile ducts, even if there are no dilatations [25, 36].

However, contrast-enhanced MRC reported an accurate visualization of bile duct anomalies also using Gd-BOPTA; 3D T1-weighted images acquired 90 minutes after Gd-BOPTA administration demonstrated a better visualization of bile duct anomalies in a population of potential liver donor, in comparison with 2D SSFSE cholangiography [37].

### 3.2. Evaluation of Biliary Cysts

Biliary cysts account for about 1% of all benign biliary diseases [38]. They are commonly in Asian population and privilege the female gender.

Generally, they are diagnosed in infants or children, even if occasionally they are detected in adults (up to 20%) [38, 39]. Their diagnosis is important for several reasons: first of all, they represent a risk for tumour development, namely, cholangiocarcinoma. In addition, other diseases and complications may occur: lithiasis, cholangitis, biliary cirrhosis, portal hypertension, and pancreatitis [38].

The cystic dilatation of the biliary tree has been classified by several authors; mainly, there are 2 classifications, edited, respectively, by Alonso-Lej in 1959 and by Todani in 1977 [40, 41].

The latest distinguishes 5 types of congenital biliary cysts [41]:

- Type I, represented by choledochal cyst
- Type II, usually represented by a diverticulum of common bile duct
- Type III, also known as choledochocele
- Type IV, which includes multiple communicating intra- and extrahepatic duct cysts
- Type V, which is called Caroli's disease

The diagnosis of intrahepatic forms should be differentiated by other conditions, such as adult polycystic liver disease or the von Meyenburg complex [25, 42].

**Figure 5:**
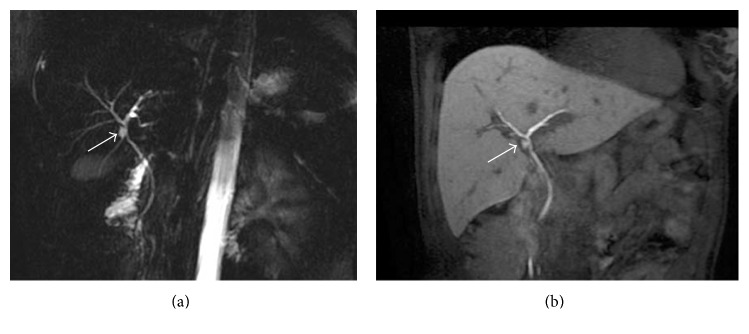
Biliary cystic disease, type II. MRCP image (a) shows a small paracholedochal cyst, with homogeneous hyperintense signal (white arrow). 3D T1-weighted spoiled gradient echo image (b)—obtained in an excretory phase—shows opacification of the cystic lesion (white arrow); a diagnosis of type II biliary cystic disease was achieved.

Hepatobiliary contrast agent excretion could be useful in differentiating choledochal cyst (Figure 5), particularly in the diagnosis of IV and V types of choledochal cyst [43]. Due to their typical morphological appearance, choledochal cyst, diverticulum, and choledochocele are generally easily diagnosed by conventional MRCP. Types IV and V mean the presence of small cysts in the hepatic parenchyma, and cholangiographic sequences—based on T2-weighted images—are not able to clarify the relation with the biliary tree.

Lee et al. [25] emphasized the diagnostic capability of a hepatobiliary contrast in the diagnosis of congenital hepatic cystic disease: therefore, gadoxetic acid-enhanced MRC shows—after 20 minutes—opacification of biliary cysts (Figure 5) if they are in communication with the biliary tree [25].

### 3.3. Biliary Injuries: Detection of Biliary Leaks

**Figure 6:**
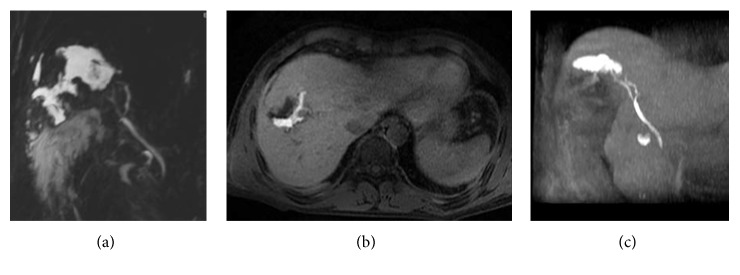
Biliary leak after hepatic resection. (a) shows a large fluid collection—hyperintense on MR cholangiography—located in the cranial part of the liver. (b) and (c)—obtained in excretory phase—show opacification of the fluid collection, suggesting a biliary injury.

Surgical injuries—which can cause irregular excretion of bile—are mainly represented by leakage, stricture, or complete transection and excision of a ductal segment, with or without obstruction of the proximal biliary tree by surgical clips [17]. Bile leak may occur after cholecystectomy [17, 44–46], liver resection (Figure 6), biliary anastomosis during liver transplantation [47], hepaticojejunal anastomosis [17], and, less frequently, abdominal traumatic lesions.

**Figure 7:**
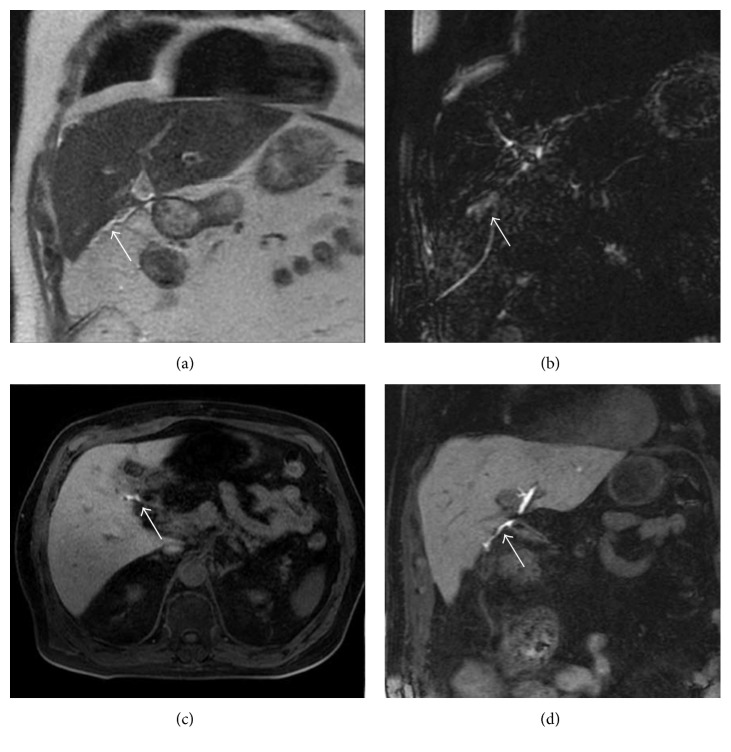
Biliary leak after cholecystectomy. Coronal T2-weighted single-shot fast spin-echo image (a) and 3D FRFSE cholangiography (b) show a small fluid collection (white arrows in (a) and (b)). Images obtained in excretory phase ((c) and (d)) show progressive opacification (white arrows): a diagnosis of biliary leak was performed.

The possibility to detect the bile leak using MRCP has many advantages: there is no ionizing exposure, in contrast to conventional cholangiography; in addition, it is less invasive, without any risk of cholangitis. However, conventional 2D and/or 3D unenhanced MRCP is limited in the evaluation of biliary leak, because it provides only a morphologic information about damage. Moreover, a biliary leak could be suspected in case of a fluid collection—hyperintense on unenhanced cholangiography sequences—contiguous to the site of surgical anastomosis or cystic duct ligation. However, a clear explanation is reached only with contrast opacification of the biliary system (Figure 7).

To increase the diagnostic accuracy of bile leak detection, conventional MRCP, based on T2-weighted acquisition, has been compared to gadoxetic acid-enhanced T1-weighted gradient echo sequences.

In the assessment of bile leak, Gd-BOPTA-enhanced MRC has been compared to “conventional bile duct opacification obtained by endoscopy or t-tube cholangiogram,” as reported by Fontarensky et al. [48]. Opacification of bile ducts was observed in absence of leak, whereas it was never reported in case of bile leak; in addition, opacification of periliver fluid collection was also observed [48]. Authors conclude that Gd-BOPTA-enhanced MRC is a noninvasive method for detecting biliary leakage.

In the paper published by Kantarcı et al. [49], biliary extravasation with progressive filling of peribiliary fluid collection by Gd-EOB-DTPA has been demonstrated; namely, this finding was retained “complementary” to the T2 imaging, which is able to demonstrate fluid collection but does not allow to detect exactly the site of “extravasation” [49].

The accuracy of the combination of conventional MRCP (based on morphological T2 sequences) and contrast-enhanced MRC was superior to T2 sequences alone: mean sensitivities were, respectively, 79% and 59%, and diagnostic accuracy was 84% and 58% [49].

Gadoxetic acid-enhanced MRC reported high sensitivity and specificity in the diagnosis of bile leak after biliary surgery [50]. The timing of the delayed acquisition—in case of gadoxetic acid-enhanced MRC—has been analyzed in literature. In a paper by Cieszanowski et al. [51], 34 patients with suspicion of bile leak were studied using Gd-EOB-DTPA. Delayed images were acquired 20–25 minutes, 60–90 minutes, and 150–180 minutes after gadoxetic acid injection [51]. The highest diagnostic accuracy—“for the diagnosis of an active bile leak”—was obtained by the combination of all dataset of images, with an overall sensitivity of 96.4%, specificity of 100%, and accuracy of 97.1%. Delayed images, obtained after 20–25 minutes, reported an overall sensitivity of 42.9%, whereas the combination of 20–25 minutes and 60–90 minutes delayed images allowed achievement of a sensitivity value of 92.9% [51]. Authors recommended the acquisition of delayed images (150–180 minutes) to identify bile leak in cases of biliary dilatation of moderate liver dysfunction.

However, gadoxetic acid-enhanced MRC is “a highly reliable technique” for the detection of bile leak after hepatobiliary surgery, even if three-dimensional T1-weighted gradient-echo sequences were acquired 20 minutes after contrast administration [52]; indeed, in a series described by Alegre Castellanos et al. [52], which included 23 patients, the diagnostic accuracy of Gd-EOB-DTPA-enhanced MR imaging was 100% in the detection or exclusion of bile leak. These results clearly show that an early phase using Gd-EOB-DTPA administration is adequate to detect bile leak after hepatobiliary surgery.

### 3.4. Biliary-Enteric Anastomosis

**Figure 8:**
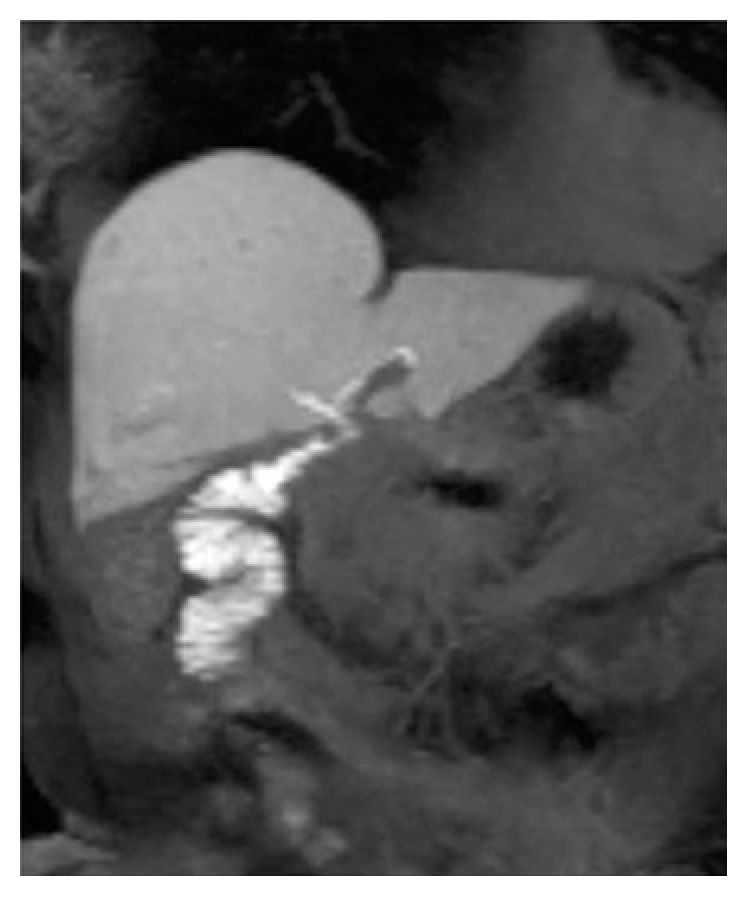
Biliary-enteric anastomosis. Excretory phase provides a clear visualization of the anastomoses.

**Figure 9:**
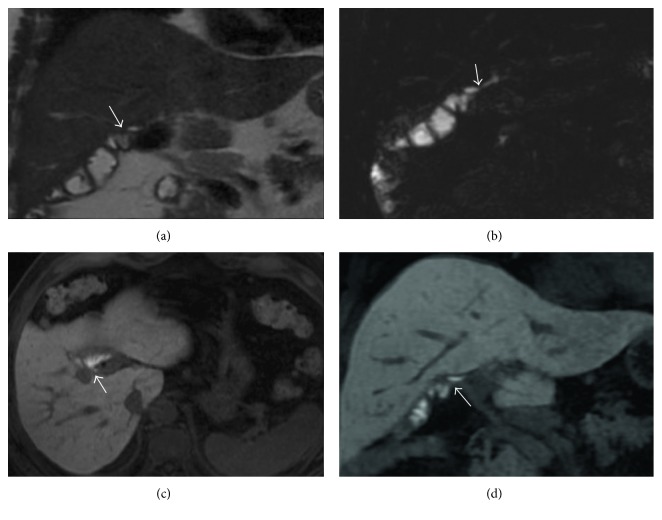
A 60-year-old patient with a biliary-enteric anastomoses. Coronal T2-weighted image (a) and 3D MRCP (b) clearly provide morphological representation of biliary-enteric anastomoses (white arrows). Contrast-enhanced MRC images show opacification of anastomoses (white arrows in (c) and (d)), adding functional information to morphology.

**Figure 10:**
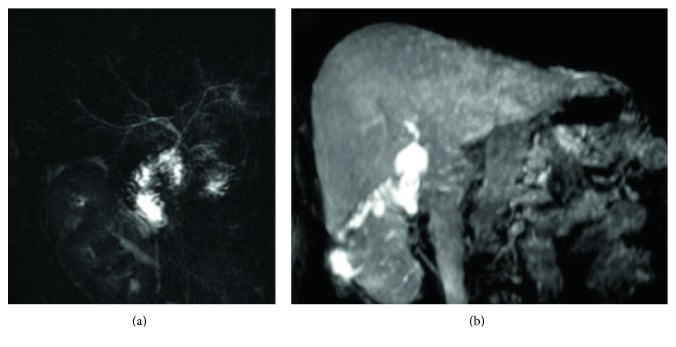
Patient with a biliary-enteric anastomosis, performed after duodenopancreatectomy. 2D T2-weighted cholangiography (a) shows normal appearance of a biliojejunal anastomosis. Excretory phase—obtained after contrast administration of Gd-BOPTA—confirms regular opacification at the surgical connection (b).

Biliary-enteric anastomoses may develop some complications, represented by anastomotic leak, stricture, hemorrhage, inflammation, and stones [53, 54]. Several papers have recently demonstrated that MRC—using intravenous administration of hepatospecific contrast—could be a valid diagnostic tool for the assessment of biliary-enteric anastomoses complications. It provides a clear visualization of the anastomoses (Figures 8–10), allowing the visualization of stones or identification of a stenosis [54]; in addition, the degree of jejunal opacification represents a valid diagnostic tool to assess the functionality of the anastomosis (Figure 10).

In the past, MRC has been used as a reliable method for the assessment of stricture or other complications in patients with biliary-enteric anastomoses [55, 56]. Hottat et al. [55] analyzed 13 patients with hepaticojejunostomy using intravenous administration of Mn-DPDP, obtaining useful information in cases with biliary dilatation observed at T2-weighted images [55]. In 1997, Pavone et al. [56] investigated 24 patients: in all cases examined, both observers correctly assessed dilatation of biliary ducts [56]. Fat-suppressed three-dimensional turbo spin-echo sequence was highly reliable, identifying bile duct irregularities in 6/8 patients with cholangitis; the authors were also able to assess the degree of stenosis in all patients with strictures, and the presence of stones in 9 out of 10 patients [56].

The diagnostic capability of MRCP using a hepatobiliary contrast agent seems to be dependent on the type of contrast medium. Kandasamy et al. [57] evaluated the role of contrast-enhanced MR imaging for the assessment of biliary enteroanastomotic stricture in a group of 21 patients; they used gadobenate dimeglumine as contrast medium [57]. For diagnosis of biliary stricture, sensitivity, specificity, positive predictive values, and negative predictive values were 94.4%, 80%, 94.4%, and 80% using conventional T2 MRCP, whereas contrast-enhanced T1-weighted MR imaging showed values of 40%, 75%, 80%, and 33.3% [57].

### 3.5. Biliary Complications after Orthotopic Liver Transplantation (OLT)

After liver transplantation, biliary complications occur commonly, ranging from 5% up to 30% of patients [58]. They are most frequently represented by bilomas, biliary leakage, and biliary strictures, of both the biliary anastomosis and the intra- and extrahepatic bile ducts. Early complications consist in biliary leakage and in nonanastomotic strictures; they are caused by thrombosis of the hepatic artery [58, 59]. Biliary strictures can often be seen several months to years after liver transplantation, and privilege anastomotic junction; in these cases, they are classified as “late complications” [59].

As previously reported, assessment of biliary leakages using heavily T2-weighted MR cholangiography is limited, due to the fact that it is based only on morphologic demonstration of fluid collections.

**Figure 11:**
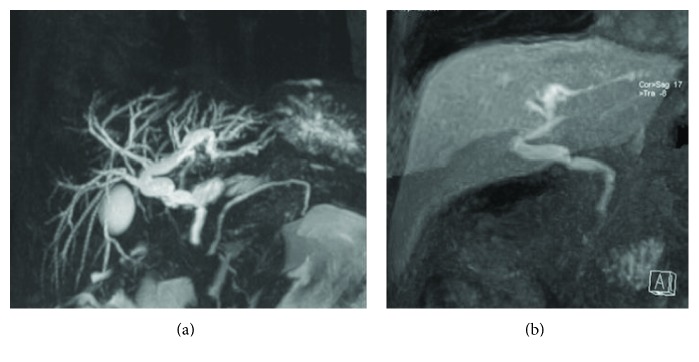
Biliary anastomosis after OLT. 3D MIP FRFSE image (a) and excretory phase (b) show a functioning choledochal anastomosis after OLT.

MRCP allows the visualization of anastomotic or nonanastomotic strictures (Figure 11), bile duct stones and papillary dysfunction [4, 60]. Degree of strictures could be also assessed on the basis of biliary tree dilatation.

However, contrast-enhanced MRC should be routinely performed in patients with high clinical suspicion of biliary complications after liver transplantation, in order to increase diagnostic accuracy in the detection of biliary leakage or biliary strictures [59].

Normal MRC findings include narrowing of the common bile duct and mild thickening of the wall, without bile duct dilatation involving the cranial segments of the biliary tree [58].

### 3.6. Cholecystitis and Gallbladder Dysfunction

Typical imaging findings of cholecystitis are represented by wall thickening, oedema, and fluid collection around the gallbladder. These findings are very often observed on ultrasonography, CT, and MR examinations. However, sometimes clinical and morphological appearances remain doubtful.

Gadoxetic acid-enhanced scans could be adopted as “functional markers” of gallbladder contraction. Several studies evaluated the kinesis of the cholecyst: generally, the wall contraction has been induced using a fatty meal or a stimulant agent [61, 62]. Exclusion of gallbladder or time range for reflux into the cystic duct—due to oedema and wall thickening—could be used as valid diagnostic tools for the diagnosis of inflammation [63].

Akpinar et al. [64] evaluated 11 consecutive patients with acute right upper quadrant pain and equivocal clinical and sonographic findings. Patients underwent gadobenate dimeglumine-enhanced MRC: on delayed images, significant enhancement was seen in 10 out of 11 patients, and gallbladder excretion was not revealed in all of them [64].

Choi et al. [65] evaluated percentages of contrast agent in gallbladder and cystic duct, which were used as markers for predicting the presence of acute cholecystitis [65]. They include patients with acute cholecystitis and chronic cholecystitis and healthy subjects, acquiring delayed (60 minutes) gadoxetic acid-enhanced MR images [65]. In their study, a cut-off value of 30% “as predictor of acute cholecystitis comparing with healthy volunteers” was found, with sensitivity of 93.8% and specificity of 100%; a cut-off value of 0% “as predictor of acute cholecystitis comparing with chronic cholecystitis” was observed, with sensitivity of 81.2% and specificity of 82.6% [65].

Diagnosis of gallbladder dyskinesia was analyzed by Lee et al. [61] calculating the ejecting fraction: in their study, the gallbladder fraction was not different both in enhanced MRC and in hepatobiliary scintigraphy in 8 out of 18 patients analyzed [61]. Values of gallbladder fraction—using gadoxetic acid—lower than 35–40% indicate dyskinesia, and these patients could benefit from surgery [61–63, 65, 66]. Gadoxetic acid-enhanced MRC could be used as an alternative to hepatobiliary scintigraphy in patients with clinical suspicion of functional biliary pain.

## 4. Conclusion

In the last years, contrast-enhanced MRC has increased diagnostic accuracy for biliary disease, allowing the addition of “functional information” or “excretory function” to conventional imaging.

Clinical indications of contrast-enhanced MRC—that have been introduced in daily routine—include the following: (a) evaluation of congenital biliary cysts; (b) detection of biliary leaks; (c) assessment of biliary-enteric anastomoses; and (d) demonstration of biliary complications after OLT. In these clinical scenarios, the diagnostic capabilities of contrast-enhanced MRC may reach high values; namely, in detection of biliary leaks and assessment of biliary-enteric anastomoses, sensitivities and diagnostic accuracy are superior to those reported by conventional MRCP based on T2 sequences alone [49, 51].

Contrast-enhanced MRC may be useful to better demonstrate the presence of biliary variants—when preoperative assessment of biliary anatomy remains doubtful after conventional MRCP; finally, it represents a valid alternative to hepatobiliary scintigraphy—for patients having clinical suspicion of gallbladder dyskinesia.

## References

[1] Wallner B. K., Schumacher K. A., Weidenmaier W., Friedrich J. M. (1991). Dilated biliary tract: evaluation with MR cholangiography with a T2-weighted contrast-enhanced fast sequence. *Radiology*.

[2] Mandarano G., Sim J. (2008). The diagnostic MRCP examination: overcoming technical challenges to ensure clinical success. *Biomedical Imaging and Intervention Journal*.

[3] Griffin N., Charles-Edwards G., Grant L. A. (2012). Magnetic resonance cholangiopancreatography: the ABC of MRCP. *Insights Imaging*.

[4] Pavone P., Laghi A., Panebianco V. (1998). Diagnosis of biliary and pancreatic ducts with magnetic resonance cholangio pancreatography (MRCP). *Saudi Journal of Gastroenterology*.

[5] Barish M. A., Yucel E. K., Soto J. A., Chuttani R., Ferrucci J. T. (1995). MR cholangiopancreatography: efficacy of three-dimensional turbo spin-echo technique. *AJR. American Journal of Roentgenology*.

[6] Aubè C., Delorme B., Yzet T. (2005). MR cholangiopancreatography versus endoscopic sonography in suspected common bile duct lithiasis: a prospective, comparative study. *AJR. American Journal of Roentgenology*.

[7] Sodickson A., Mortele K. J., Barish M. A., Zou K. H., Thibodeau S., Tempany C. M. (2006). Three-dimensional fast-recovery fast spin-echo MRCP: comparison with two-dimensional single-shot fast spin-echo techniques. *Radiology*.

[8] Vitellas K. M., Keogan M. T., Spritzer C. E., Nelson R. C. (2000). MR cholangiopancreatography of bile and pancreatic duct abnormalities with emphasis on the single-shot fast spin-echo technique. *Radiographics*.

[9] Dooms C. G., Fisher M. R., Higgins C. B., Hricak H., Goldberg H. I., Margulis A. R. (1986). MR imaging of dilated biliary tract. *Radiology*.

[10] Dooms G. C., Kerlan R. K., Hricak H., Wall S. D., Margulis A. R. (1986). Cholangiocarcinoma: imaging by MR. *Radiology*.

[11] Fulcher A. S., Turner M. A., Capps G. W. (1999). MR cholangiography: technical advances and clinical applications. *Radiographics*.

[12] Palmucci S., Mauro L. A., Coppolino M. (2010). Evaluation of the biliary and pancreatic system with 2D SSFSE, breathhold 3D FRFSE and respiratory-triggered 3D FRFSE sequences. *La Radiologia Medica*.

[13] Semelka R. C., Shoenut J. P., Kroeker M. A. (1992). Bile duct disease: prospective comparison of ERCP, CT, and fat suppression MRI. *Gastrointestinal Radiology*.

[14] Park M. S., Kim T. K., Kim K. W. (2004). Differentiation of extrahepatic bile duct cholangiocarcinoma from benign stricture: findings at MRCP versus ERCP. *Radiology*.

[15] Park D. H., Kim M. H., Lee S. S. (2004). Accuracy of magnetic resonance cholangiopancreatography for locating hepatolithiasis and detecting accompanying biliary strictures. *Endoscopy*.

[16] Girometti R., Brondani G., Cereser L. (2010). Post-cholecystectomy syndrome: spectrum of biliary findings at magnetic resonance cholangiopancreatography. *The British Journal of Radiology*.

[17] Hoeffel C., Azizi L., Lewin M. (2006). Normal and pathologic features of the postoperative biliary tract at 3D MR cholangiopancreatography and MR imaging. *Radiographics*.

[18] Laurent V., Ayav A., Hoeffel C. (2009). Imaging of the postoperative biliary tract. *Journal de Radiologie*.

[19] Weinmann H. J., Schuhmann-Giampieri G., Schmitt-Willich H., Vogler H., Frenzel T., Gries H. (1991). A new lipophilic gadolinium chelate as a tissue-specific contrast medium for MRI. *Magnetic Resonance in Medicine*.

[20] Schuhmann-Giampieri G., Schmitt-Willich H., Press W. R., Negishi C., Weinmann H. J., Speck U. (1992). Preclinical evaluation of Gd-EOB-DTPA as a contrast agent in MR imaging of the hepatobiliary system. *Radiology*.

[21] Clement O., Muhler A., Vexler V., Berthezene Y., Brasch R. C. (1992). Gadolinium-ethoxybenzyl-DTPA, a new liver-specific magnetic resonance contrast agent. Kinetic and enhancement patterns in normal and cholestatic rats. *Investigative Radiology*.

[22] Hamm B., Vogl T. J., Branding G. (1992). Focal liver lesions: MR imaging with Mn-DPDP – initial clinical results in 40 patients. *Radiology*.

[23] Kopp A. F., Laniado M., Aicher K. P., Grönewäller E. F., Claussen C. D. (1992). Manganese DPDP as a contrast medium for MR tomography of focal liver lesions. Tolerance and image quality in 20 patients. *Rofo*.

[24] Vogl T. J., Pegios W., Waitzinger J., Pirovano G., Balzer J., Lissner J. (1992). NMR tomography of the liver with the new contrast medium Gd-BOPTA. The results of an in-vivo phase-I test. *Rofo*.

[25] Lee N. K., Kim S., Lee J. W. (2009). Biliary MR imaging with Gd-EOB-DTPA and its clinical applications. *Radiographics*.

[26] Nagle S. K., Busse R. F., Brau A. C. (2009). High-resolution free-breathing 3D T1 weighted hepatobiliary imaging optimized for Gd-EOB-DTPA. *Proceedings on International Society for Magnetic Resonance in Medicine*.

[27] Kirchin M. A., Pirovano G. P., Spinazzi A. (1998). Gadobenate dimeglumine (Gd-BOPTA): an overview. *Investigative Radiology*.

[28] Frydrychowicz A., Lubner M. G., Brown J. J. (2012). Hepatobiliary MR imaging with gadolinium-based contrast agents. *Journal of Magnetic Resonance Imaging*.

[29] Cavagna F. M., Maggioni F., Castelli P. M. (1997). Gadolinium chelates with weak binding to serum proteins. A new class of high-efficiency, general purpose contrast agents for magnetic resonance imaging. *Investigative Radiology*.

[30] Palmucci S. (2014). Focal liver lesions detection and characterization: the advantages of gadoxetic acid-enhanced liver MRI. *World Journal of Hepatology*.

[31] Stelter L., Freyhardt P., Grieser C. (2014). An increased flip angle in late phase Gd-EOB-DTPA MRI shows improved performance in bile duct visualization compared to T2w-MRCP. *European Journal of Radiology*.

[32] Stelter L., Grieser C., Fernándes C. M. (2012). Flip angle modulations in late phase Gd-EOB-DTPA MRI improve the identification of the biliary system. *European Journal of Radiology*.

[33] Frydrychowicz A., Nagle S. K., D'Souza S. L., Vigen K. K., Reeder S. B. (2011). Optimized high-resolution contrast-enhanced hepatobiliary imaging at 3 tesla: a cross-over comparison of gadobenate dimeglumine and gadoxetic acid. *Journal of Magnetic Resonance Imaging*.

[34] Hyodo T., Kumano S., Kushihata F. (2012). CT and MR cholangiography: advantages and pitfalls in perioperative evaluation of biliary tree. *The British Journal of Radiology*.

[35] Seale M. K., Catalano O. A., Saini S., Hahn P. F., Sahani D. V. (2009). Hepatobiliary-specific MR contrast agents: role in imaging the liver and biliary tree. *Radiographics*.

[36] Carlos R. C., Hussain H. K., Song J. H., Francis I. R. (2002). Gadolinium-ethoxybenzyl-diethylenetriamine pentaacetic acid as an intrabiliary contrast agent: preliminary assessment. *AJR. American Journal of Roentgenology*.

[37] Napolitano M., Catalano O. A., Monti C., Leni D., Colombo E., Vanzulli A. (2003). *SSFSE- vs Gd-BOPTA-Enhanced MR Cholangiography in the Detection of Bile Ducts Anomalies in Adult Living Liver Donors (SS)*.

[38] Jabłońska B. (2012). Biliary cysts: etiology, diagnosis and management. *World Journal of Gastroenterology*.

[39] Singham J., Yoshida E. M., Scudamore C. H. (2009). Choledochal cysts: part 1 of 3: classification and pathogenesis. *Canadian Journal of Surgery*.

[40] Alonso-Lej F., Rever W. B., Pessagno D. J. (1959). Congenital choledochal cyst, with a report of 2, and an analysis of 94, cases. *International Abstracts of Surgery*.

[41] Todani T., Watanabe Y., Narusue M., Tabuchi K., Okajima K. (1977). Congenital bile duct cysts: classification, operative procedures, and review of thirty-seven cases including cancer arising from choledochal cyst. *American Journal of Surgery*.

[42] Vachha B., Sun M. R., Siewert B., Eisenberg R. L. (2011). Cystic lesions of the liver. *AJR. American Journal of Roentgenology*.

[43] Gupta R. T., Brady C. M., Lotz J., Boll D. T., Merkle E. M. (2010). Dynamic MR imaging of the biliary system using hepatocyte-specific contrast agents. *AJR. American Journal of Roentgenology*.

[44] Ernst O., Sergent G., Mizrahi D., Delemazure O., L'Herminé C. (1999). Biliary leaks: treatment by means of percutaneous transhepatic biliary drainage. *Radiology*.

[45] Branum G., Schmitt C., Baillie J. (1993). Management of major biliary complications after laparoscopic cholecystectomy. *Annals of Surgery*.

[46] Wright T. B., Bertino R. B., Bishop A. F. (1993). Complications of laparoscopic cholecystectomy and their interventional radiologic management. *Radiographics*.

[47] Sherman S., Jamidar P., Shaked A., Kendall B. J., Goldstein L. I., Busuttil R. W. (1995). Biliary tract complications after orthotopic liver transplantation. Endoscopic approach to diagnosis and therapy. *Transplantation*.

[48] Fontarensky M., Montoriol P. F., Buc E., Poincloux L., Petitcolin V., Da Ines D. (2013). Advantages of gadobenate dimeglumine-enhanced MR cholangiography in the diagnosis of post-liver transplant bile leakage. *Diagnostic and Interventional Imaging*.

[49] Kantarcı M., Pirimoglu B., Karabulut N. (2013). Non-invasive detection of biliary leaks using Gd-EOB-DTPA-enhanced MR cholangiography: comparison with T2-weighted MR cholangiography. *European Radiology*.

[50] Ratcliffe G. E., Kirkpatrick I. D., Anik Sahni V. (2014). Detection and localization of bile duct leaks after cholecystectomy using Gd-EOB-DTPA-enhanced MR cholangiography: retrospective study of 16 patients. *Journal of Computer Assisted Tomography*.

[51] Cieszanowski A., Stadnik A., Lezak A. (2013). Detection of active bile leak with Gd-EOB-DTPA enhanced MR cholangiography: comparison of 20-25 min delayed and 60-180 min delayed images. *European Journal of Radiology*.

[52] Alegre Castellanos A., Molina Granados J. F., Escribano Fernandez J., Gallardo Muñoz I., Triviño Tarradas Fde A. (2012). Early phase detection of bile leak after hepatobiliary surgery: value of Gd-EOB-DTPA-enhanced MR cholangiography. *Abdominal Imaging*.

[53] Zafar S. N., Khan M. R., Raza R. (2011). Early complications after biliary enteric anastomosis for benign diseases: a retrospective analysis. *BMC Surgery*.

[54] Boraschi P., Donati F. (2013). Biliary-enteric anastomoses: spectrum of findings on Gd-EOB-DTPA-enhanced MR cholangiography. *Abdominal Imaging*.

[55] Hottat N., Winant C., Metens T., Bourgeois N., Devière J., Matos C. (2005). MR cholangiography with manganese dipyridoxyl diphosphate in the evaluation of biliary-enteric anastomoses: preliminary experience. *AJR. American Journal of Roentgenology*.

[56] Pavone P., Laghi A., Catalano C. (1997). MR cholangiography in the examination of patients with biliary-enteric anastomoses. *AJR. American Journal of Roentgenology*.

[57] Kandasamy D., Sharma R., Seith Bhalla A. (2011). MR evaluation of biliary-enteric anastomotic stricture: does contrast-enhanced T1W MRC provide additional information?. *Clinics and Research in Hepatology and Gastroenterology*.

[58] Girometti R., Como G., Bazzocchi M., Zuiani C. (2014). Post-operative imaging in liver transplantation: state-of-the-art and future perspectives. *World Journal of Gastroenterology*.

[59] Girometti R., Cereser L., Como G., Zuiani C., Bazzocchi M. (2008). Biliary complications after orthotopic liver transplantation: MRCP findings. *Abdominal Imaging*.

[60] Fulcher A. S., Turner M. A. (1999). Orthotopic liver transplantation: evaluation with MR cholangiography. *Radiology*.

[61] Lee J. K., Kim Y., Lee S., Park J. E. (2015). Hepatobiliary phase of gadoxetic acid-enhanced MR in patients suspected of having gallbladder dyskinesia: comparison with hepatobiliary scintigraphy. *Clinical Imaging*.

[62] Lee J. K., Kwag E., Kim J. S. (2012). *Comparison of the hepatobiliary phase of gadoxetic acid-enhanced MR and hepatobiliary scintigraphy for the evaluation of patients suspected of having acute cholecystitis or gallbladder dyskinesia: a preliminary study*.

[63] Krishnan P., Gupta R. T., Boll D. T., Brady C. M., Husarik D. B., Merkle E. M. (2012). Functional evaluation of cystic duct patency with Gd-EOB-DTPA MR imaging: an alternative to hepatobiliary scintigraphy for diagnosis of acute cholecystitis?. *Abdominal Imaging*.

[64] Akpinar E., Turkbey B., Karcaaltincaba M. (2009). Initial experience on utility of gadobenate dimeglumine (Gd-BOPTA) enhanced T1-weighted MR cholangiography in diagnosis of acute cholecystitis. *Journal of Magnetic Resonance Imaging*.

[65] Choi I. Y., Cha S. H., Yeom S. K. (2014). Diagnosis of acute cholecystitis: value of contrast agent in the gallbladder and cystic duct on Gd-EOB-DTPA enhanced MR cholangiography. *Clinical Imaging*.

[66] Vassiliou M. C., Laycock W. S. (2008). Biliary dyskinesia. *Surgical Clinics of North America*.

